# CD40L Expression Allows CD8^+^ T Cells to Promote Their Own Expansion and Differentiation through Dendritic Cells

**DOI:** 10.3389/fimmu.2017.01484

**Published:** 2017-11-06

**Authors:** Neil Q. Tay, Debbie C. P. Lee, Yen Leong Chua, Nayana Prabhu, Nicholas R. J. Gascoigne, David M. Kemeny

**Affiliations:** ^1^NUS Graduate School for Integrative Sciences and Engineering, National University of Singapore, Singapore, Singapore; ^2^Department of Microbiology and Immunology, Yong Loo Lin School of Medicine, National University of Singapore, Singapore, Singapore; ^3^Immunology Programme, Life Sciences Institute, National University of Singapore, Singapore, Singapore

**Keywords:** CD8^+^ T cells, CD40, CD40L, dendritic cells, cell proliferation, cell differentiation, influenza, *Listeria monocytogenes*

## Abstract

CD8^+^ T cells play an important role in providing protective immunity against a wide range of pathogens, and a number of different factors control their activation. Although CD40L-mediated CD40 licensing of dendritic cells (DCs) by CD4^+^ T cells is known to be necessary for the generation of a robust CD8^+^ T cell response, the contribution of CD8^+^ T cell-expressed CD40L on DC licensing is less clear. We have previously shown that CD8^+^ T cells are able to induce the production of IL-12 p70 by DCs in a CD40L-dependent manner, providing some evidence that CD8^+^ T cell-mediated activation of DCs is possible. To better understand the role of CD40L on CD8^+^ T cell responses, we generated and characterized CD40L-expressing CD8^+^ T cells both *in vitro* and *in vivo*. We found that CD40L was expressed on 30–50% of effector CD8^+^ T cells when stimulated and that this expression was transient. The expression of CD40L on CD8^+^ T cells promoted the proliferation and differentiation of both the CD40L-expressing CD8^+^ T cells and the bystander effector CD8^+^ T cells. This process occurred *via* a cell-extrinsic manner and was mediated by DCs. These data demonstrate the existence of a mechanism where CD8^+^ T cells and DCs cooperate to maximize CD8^+^ T cell responses.

## Introduction

CD40L, or CD154, is canonically expressed on CD4^+^ T cells and it is a principal modulator of a wide range of humoral and cellular immune responses (1, 2). One of the primary functions of CD40L is the T cell-mediated activation of DCs and monocytes (3, 4), in a process known as DC licensing. For DCs to properly prime CD8^+^ T cell responses, licensing *via* DC-expressed CD40 is required (5–7). CD4^+^ T cell help has been identified as one of the sources of CD40L in the priming of CD8^+^ T cell responses and the CD4^+^ cells provide help by signaling either *via* DCs or to CD8^+^ T cells directly (8–10). Although several studies involving CD40L^−/−^ mice have shown that CD40 licensing is not required to mount a strong primary CD8^+^ T cell response in response to viruses such as the lymphocytic choriomeningitis virus, lack of CD40L results in lower numbers of memory CD8^+^ T cells (11). Since the generation of protective memory CD8^+^ T cells is a hallmark of an effective CD8^+^ T cell response, these studies collectively show that CD40L plays a central role in CD8^+^ T cell immunity.

Despite the conventional association of CD40L expression with CD4^+^ T cells, other data suggest that CD8^+^ T cells are also capable of expressing CD40L (8, 12–15) and this may confer on CD8^+^ T cells the ability to regulate antigen-specific immune responses. The expression of CD40L on CD8^+^ T cells has also been hypothesized to allow CD8^+^ T cells to provide autocrine T cell help in the absence of CD4^+^ T helper cells (16). We have previously demonstrated that CD40–CD40L interaction is necessary for activated CD8^+^ T cells to prime DCs for IL-12 production in an antigen-specific manner (13, 17). The expression of CD40L on CD8^+^ T cells also appears to be transient and to require activation through the TCR, which involves the formation of the supramolecular activation complex at the immunological synapse (18) where TCRs, costimulatory molecules, and Src-family kinases localize. Currently, there is some evidence that the expression of CD40 on CD8^+^ T cells is important in some models of CD8^+^ T cell activation (8, 19) and since CD40L is expressed on CD8^+^ T cells themselves, it may be possible that CD8^+^ T cell–T cell interactions involving CD40–CD40L may be relevant. However, it remains unclear whether the expression of CD40L on CD8^+^ T cells serves predominantly to fulfill this role, whether it serves similar functions as when it is expressed on CD4^+^ T cells, and what are the relevant target cell types of CD8^+^ T cell-mediated CD40–CD40L interactions.

Although we know that CD8^+^ T cells can express CD40L, it is unknown whether CD8^+^ T cell-expressed CD40L is able to license DCs and contribute to CD8^+^ T cell responses in a semi-autocrine manner. Autocrine signaling often results in some form of positive feedback which in turn allows immune responses to quickly amplify themselves, and there is evidence that such mechanisms are present in CD8^+^ T cells. For example, during secondary CD8^+^ T cell responses, autocrine IL-2 mediates the expansion of CD8^+^ memory T cells (20). Therefore, we hypothesize that one of the roles of CD40L on CD8^+^ T cells is to allow them to signal *via* DCs as part of a positive-feedback loop. Recent studies by Shugart and coworkers provided support to this “self-help” hypothesis by showing that the expression of CD40L on CD8^+^ T cells is a determinant of secondary expansion in CD40^−/−^ mice (15) although the exact mechanisms by which this occurs remain unclear. Here, we showed that CD8^+^ T cells that express CD40L can promote their own expansion *via* the activation of DCs.

## Materials and Methods

### Mice

Sex- and age-matched mice were used in all experiments. C57BL/6J mice were bred at the Department of Comparative Medicine, National University of Singapore. OT-I mice were obtained from Charles River Laboratories. CD40L^−/−^ (B6.129S2-Cd40lg^tm1Imx^/J) and C57BL/6J CD45.1^+^ (B6.SJL-Ptprc^a^ Pepc^b^/BoyJ) mice were purchased from Jackson Laboratory. OT-I CD40L^−/−^ mice were generated by crossbreeding of OT-I mice with CD40L^−/−^ mice. All mice were maintained in specific pathogen free conditions and experiments involving live pathogens were performed in an animal biosafety level 2 facility. All experiments were conducted in accordance with institutional guidelines and were approved by the Institutional Animal Care and Use Committee.

### Bacteria and Viruses

Recombinant chicken ovalbumin secreting *Listeria monocytogenes* (Lm-OVA) was a kind gift from Dr. H. Shen (University of Pennsylvania). Bacteria were grown in brain–heart infusion (BHI) medium containing 5 µg/mL erythromycin.

Influenza virus A/PR/8/34 (PR8) was purchased from the American Type Culture Collection. Recombinant influenza A/PR/8/34 virus containing the chicken ovalbumin epitope SIINFEKL (PR8-OVA) was a gift from Paul Thomas (St. Jude Children’s Research Hospital). The viruses were propagated in 10-day old embryonated chicken eggs as previously described (21).

### Antibodies and Flow Cytometry

Antibodies to CD3 (17A2), CD3ε (145-2C11), CD4 (RM4-4, GK1.5), CD8α (53-6.7), CD25 (PC61), CD40L (MR1), CD43 (1B11), CD44 (IM7), CD45.1 (A20), CD45.2 (104), CD62L (MEL-14), CD127 (SB/199), KLRG1 (2F1/KLRG1), and IFN-γ (XMG1.2) were purchased from BD Biosciences, BioLegend, or eBioscience. All antibodies were used at a dilution of 1:100 to 1:200. H-2K^b^/OVA_257–364_ pentamers were purchased from ProImmune and used as per the manufacturer’s protocol.

For intracellular staining of cytokines and CD40L, cells were restimulated *in vitro* with 1 µg/mL OVA_257–364_ peptide (SIINFEKL) in the presence of protein transport inhibitor brefeldin A (BD GolgiPlug, BD Biosciences) for 4 h and then fixed and permeabilized using eBioscience Foxp3/transcription factor staining buffer kit (eBioscience). Flow cytometry was performed using BD LSRFortessa X-20, and the data were analyzed with FlowJo software. Cell sorting was done using Sony Sy3200 cell sorter.

### Bone Marrow-Derived Dendritic Cells (DCs)

Bone marrow cells were harvested from the tibias and femurs of C57BL/6 mice and cultured for 6 days in the presence of 400 U/mL recombinant GM-CSF. The GM-CSF differentiated DCs were pulsed with 1 µg/mL of the relevant peptides for 1 h before use.

### T Cell Isolation and Adoptive Transfer

Single cell suspensions were obtained by passing spleens from OT-I or OT-I CD40L^−/−^ mice through a 60 µm nylon mesh. Erythrocytes were lysed with ACK lysis buffer and CD8^+^ T cells were isolated either using a magnetic CD8^+^ T cell isolation kit (STEMCELL Technologies) or with fluorescence activated cell sorting. For adoptive transfer, the cells were resuspended in Hank’s balanced salt solution, and 100 µL of cell suspension containing the required number of cells was injected *via* the retro-orbital route into mice under isoflurane anesthesia.

### Infections

All infections of mice were carried out under isoflurane anesthesia. For infections with influenza A, mice were inoculated intranasally with 20 µL of phosphate-buffered saline (PBS) containing the required dose of virus. For infections with *L. monocytogenes*, 200 µL of PBS containing ~10^4^ CFU of Lm-OVA was injected retro-orbitally.

### T Cell Proliferation Assays

CD8^+^ T cells were isolated as described above and labeled with 5 µM CellTrace Violet (CTV) (Thermo Fisher Scientific) according to the manufacturer’s protocol. For *in vivo* proliferation assays, labeled cells were resuspended in HBSS, and 100 µL of cell suspension was injected *via* the retro-orbital route into anesthetized mice.

To obtain CD11c^+^ DCs for *in vitro* proliferation assays, spleens were removed from mice and digested in Liberase TL (Roche) for 30 min, and single cell suspensions were obtained by passing the digested spleens through a 60 µm nylon mesh. Magnetic selection of CD11c^+^ DCs was then performed using EasySep Mouse CD11c positive selection kit (STEMCELL Technologies), and the purified CD11c^+^ DCs were pulsed with 1 µg/mL OVA_257–264_ at 37°C for 1 h. The DCs were washed three times with fresh medium and cocultured with labeled CD8^+^ T cells at a ratio of 1 DC:10 T cells for 72 h at 37°C, 5% CO_2_.

Proliferation index was obtained using FlowJo V10 software.

### Overexpression of CD40L by Lentiviral Transduction

The full coding sequence of mouse *Cd40lg* was generated *via* PCR amplification of cDNA obtained from mouse CD8^+^ T cells, fused with mRuby3 (plasmid containing mRuby3 was a kind gift from Dr. Michael Lin, Stanford University) (22), and cloned into a lentiviral transfer vector. Lentivirus packaging was performed in 293 T cells, and virus-containing supernatant was harvested and used to transduce OT-I T hybridoma cells.

### Statistical Analysis

Statistical analyses were performed with Student’s *t*-test or analysis of variance with *post hoc* Bonferroni’s corrections. Unless stated otherwise, data are presented represent mean ± SEM with *p* values represented by *p < 0.05, **p < 0.01, ***p < 0.001, or ****p < 0.0001.

## Results

### A Subset of Effector CD8^+^ T Cells Can Express CD40L upon Stimulation

**Figure 1:**
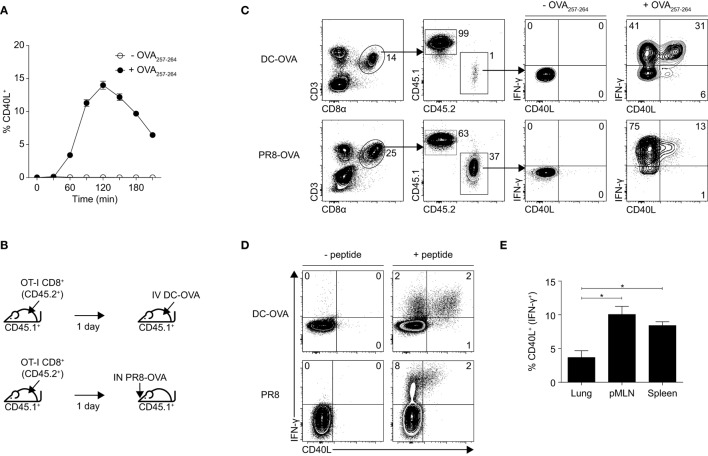
Effector CD8^+^ T cells express CD40L upon stimulation. **(A)** Effector OT-I CD8^+^ T cells were generated *in vitro* by culturing whole splenocytes with 10 ng/mL OVA_257–264_ peptide and 10 U/mL recombinant IL-2 for 3 days. Percentage of *in vitro* activated OT-I CD8^+^ T cells expressing CD40L after restimulation with 1 µg/mL OVA_257–264_ peptide was determined by flow cytometry. Data shown as mean ± SEM. **(B,C)** C57BL/6 mice (CD45.1^+^) that received 10^6^ naïve OT-I CD8^+^ T cells (CD45.2^+^) were immunized with 5 × 10^5^ dendritic cell (DC)-OVA or infected with 50 PFU of PR8-OVA the following day. **(C)** Splenocytes from the mice were restimulated *ex vivo* with 1 µg/mL OVA_257–264_ peptide 6 days after immunization (DC-OVA) or 11 days after infection (PR8-OVA) for 4 h, and the expression of IFN-γ and CD40L was determined by flow cytometry. **(D)** C57BL/6 mice were immunized with 5 × 10^5^ DC-OVA or infected with 10 PFU of PR8, and their splenocytes were restimulated *ex vivo* with 1 µg/mL OVA_257–264_ peptide 6 days after immunization (DC-OVA) or with 1 µg/mL NP_366–374_ peptide 11 days after infection (PR8) for 4 h, and the expression of IFN-γ and CD40L was determined by flow cytometry. **(E)** The number of CD40L-expressing cells in the lung, posterior mediastinal lymph node, and spleen of PR8-infected mice were compared and expressed as a percentage of all CD8^+^ T cells (top) and as a percentage of IFN-expressing CD8^+^ T cells (bottom). Data shown as mean ± SEM; analyzed with two-sided Student’s *t*-test. Data are representative of two to four independent experiments (*p < 0.05).

OT-I TCR transgenic CD8^+^ T cells that were pre-activated *in vitro* with OVA_257–264_ peptide and IL-2 were capable of transient expression of surface CD40L when restimulated with their cognate peptide OVA_257–264_ (Figure 1A). However, whether they can do so *in vivo* and also whether they can express preformed CD40L in cytosolic vesicles like in some CD4^+^ T cells (23–25) remain unclear. To investigate this, we adoptively transferred naïve CD8^+^ T cells that were isolated from OT-I mice (CD45.2^+^) into wild-type C57BL/6 mice (CD45.1^+^) and then either immunized these mice with OVA_257–264_ peptide-pulsed DCs (DC-OVA) or infected them with a strain of H1N1 influenza A that expresses the OVA_257–264_ epitope (PR8-OVA; Figure 1B). Spleens were harvested from mice immunized with DC-OVA and lungs from mice infected with PR8-OVA after 6 and 11 days, respectively, which represent the respective peaks of the CD8^+^ T cell response (data not shown). The cells were then cultured *ex vivo* with or without OVA_257–264_ peptide in the presence of brefeldin A and the ability of the adoptively transferred CD8^+^ T cells (CD45.2^+^) to produce IFN-γ and upregulate CD40L was evaluated using intracellular staining and flow cytometry. Without OVA_257–264_ stimulation, expression of IFN-γ or CD40L was not detected in the transferred OT-I CD8^+^ T cells (Figure 1C), suggesting that the analyzed cells did not constitutively express and store preformed cytosolic CD40L. Like CD8^+^ T cells that were activated *in vitro* using OVA_257–264_ peptide and IL-2, OT-I CD8^+^ T cells that were activated using these *in vivo* methods were also capable of expressing CD40L upon peptide stimulation (Figure 1C). Interestingly, the proportion of CD40L-expressing CD8^+^ T cells was lower in the PR8-OVA-infected mice compared with those in DC-OVA-immunized mice.

To ensure that the CD40L expression observed here is not an artifact of the OT-I transgenic system, we repeated the experiment without the transfer of OT-I CD8^+^ T cells. Although the number of antigen-specific CD8^+^ T cells that were generated was lower than when the mice received OT-I CD8^+^ T cells (data not shown), the CD8^+^ T cells retained the capacity to upregulate CD40L when restimulated *ex vivo* with their cognate peptide (either OVA_257–264_ for mice that were immunized with DC-OVA or NP_366–374_ for mice that were infected with PR8; Figure 1D). The expression of CD40L appeared to be dependent on TCR stimulation as unstimulated CD8^+^ T cells do not express any CD40L (Figures 1C,D). For mice that have been infected with PR8, we also compared the proportion of CD40L-expressing CD8^+^ T cells in the lung, mediastinal lymph node, and spleen of the mice and found that CD8^+^ T cells that were localized in the lymph node and the spleen expressed higher amounts of CD40L as compared with those found in the lung, with the expression being the highest on the lymph node residents (Figure 1E). This suggests a bias in the localization of CD40L-expressing CD8^+^ T cells to secondary lymphoid organs. We attempted to characterize the CD40L-expressing subset of CD8^+^ T cells using a set of surface phenotypic markers but unfortunately, could not detect any differences between the CD40L-expressing and non-expressing CD8^+^ T cells using these markers (Figure S1 in Supplementary Material). Together, these data suggest that effector CD8^+^ T cells comprise a yet unidentified subset of CD40L-expressing cells and that the expression of CD40L on these cells may play a role in CD8^+^ T cell responses, especially in the secondary lymphoid organs.

### CD8^+^ T Cell-Expressed CD40L Confers Protection against Influenza Infection

**Figure 2:**
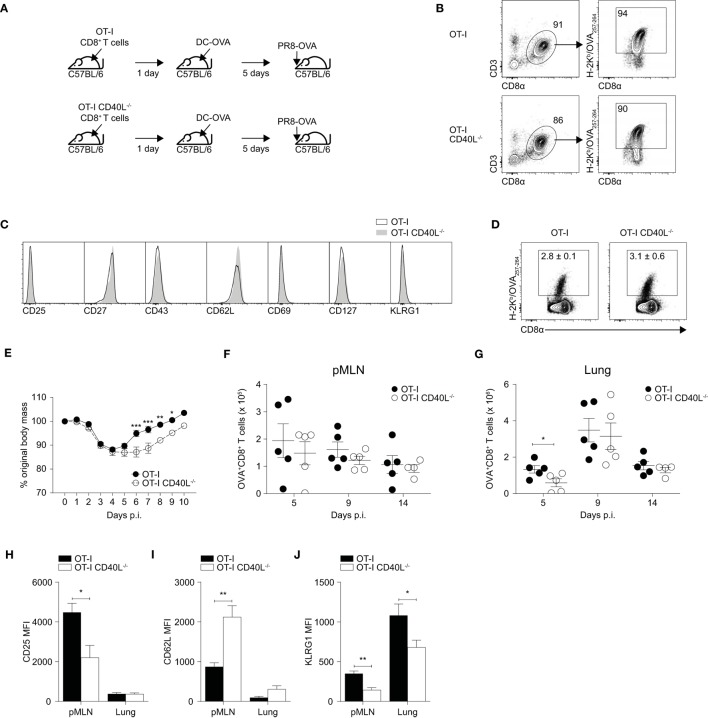
Expression of CD40L on CD8^+^ T cells confers protection against influenza A in a prime-infect model. **(A–J)** 10^5^ naïve CD8^+^ T cells were isolated from spleens of OT-I or OT-I CD40L^−/−^ mice by magnetic separation and adoptively transferred into C57BL/6 recipients. The mice were primed the following day with 5 × 10^5^ dendritic cell (DC)-OVA and infected with 50 PFU of PR8-OVA 5 days after DC priming. Antigen specificity and phenotype of purified naive CD8^+^ T cells were analyzed by flow cytometry with **(B)** H-2K^b^/OVA_257–264_ pentamers and **(C)** antibodies against CD25, CD27, CD43, CD62L, CD69, CD127, and KLRG1. **(D)** Flow cytometry was used to determine the proportion of OVA-specific CD8^+^ T cells 5 days after DC-OVA priming. **(E)** Body mass of the mice up to 10 days after infection shown as a percentage of their original mass. **(F–J)** On days 5, 9, and 14 after infection, the OVA-specific CD8^+^ T cells in the **(F)** pMLN and **(G)** lung were enumerated, and their surface expression of **(H)** CD25, **(I)** CD62L, **(J)**, and KLRG1 was determined by flow cytometry. Data are representative of two experiments and shown as mean ± SEM (*n* = 4–5 mice per group per time point) where applicable. Statistical analyses were performed using **(E)** two-way analysis of variance with Bonferroni’s correction for multiple comparisons or **(F–J)** Student’s *t*-test (*p < 0.05, **p < 0.01, ***p < 0.001). pMLN, posterior mediastinal lymph node; MFI, mean fluorescence intensity.

Although the role of CD40–CD40L signaling on various immune responses has been relatively well studied, the role of CD40L when expressed on CD8^+^ T cells remains unclear. To study the impact of CD40L deficiency more specifically when it is limited to CD8^+^ T cells, we crossed OT-I mice with CD40L^−/−^ mice and utilized a prime-infect system (Figure 2A) to investigate whether CD8^+^ T cell-expressed CD40L has any relevance in the development of influenza-specific CD8^+^ T cell responses. This system was chosen since close to half of the IFN-γ-producing CD8^+^ T cells generated by DC-OVA immunization upregulated CD40L upon restimulation (see Figure 1C) and hence, this system provides a substantial number of CD40L-expressing CD8^+^ T cells.

We first isolated naïve CD8^+^ T cells from the spleens of OT-I and OT-I CD40L^−/−^ mice using magnetic-activated cell sorting and compared both the frequency of OVA-specific CD8^+^ T cells using a fluorescent H-2K^b^ pentamer (Figure 2B) and the phenotype of the OVA-specific cells (Figure 2C). Both the frequency and phenotype of the OVA-specific CD8^+^ T cells appeared to be comparable between the OT-I and OT-I CD40L^−/−^ mice. Next, we adoptively transferred naïve CD8^+^ T cells isolated from either OT-I or OT-I CD40L^−/−^ mice into wild-type C57BL/6 mice and then immunized them with DC-OVA the next day. The resulting CD8^+^ T cell response was dominated by the transferred cells and did not appear to be dependent on the ability of the transferred cells to express CD40L, as suggested by the frequency (Figure 2D) and phenotype (not shown) of the OVA-specific CD8^+^ T cells.

We then infected the mice with PR8-OVA 5 days after they received DC-OVA and monitored them daily for changes in body mass. Adoptively transferred CD8^+^ T primed with DC-OVA conferred greater protection against influenza infection as shown by their earlier recovery of body mass that started on day 5 after infection (Figure 2E) in contrast to the usual day 8 after infection for naïve mice that did not receive adoptively transferred T cells (Figure S2 in Supplementary Material). However, the mice that received CD40L-deficient CD8^+^ T cells had a delayed recovery compared with mice that received CD40L-competent CD8^+^ T cells (Figure 2E). OVA-specific CD8^+^ T cells in the mediastinal lymph node and lung were enumerated on days 5, 9, and 14 after infection (Figures 2F,G). Although the number of OVA-specific CD8^+^ T cells present in the lymph node was consistent across both groups, there was a slight increase in number in the lungs of the mice on day 5 after infection in the group that received CD40L-competent OT-I CD8^+^ T cells, suggesting an earlier trafficking of CD8^+^ T cells to the site of infection.

To determine whether this increase in CD8^+^ T cell numbers could be due to differences in activation and differentiation, we looked at the expression of CD25, CD62L, and KLRG1 on the OVA-specific CD8^+^ T cells on day 5 after infection. We found that the CD40L-competent cells in the mediastinal lymph node expressed higher amounts of CD25 and KLRG1 and showed lower staining for CD62L compared with their CD40L-deficient counterparts (Figures 2H–J). This suggests that the CD40L-competent CD8^+^ T cells were undergoing stronger activation on day 5 after infection as indicated by the upregulation of CD25 and downregulation of CD62L, and possibly earlier trafficking to the lung after activation. Taken together, our results indicate that CD40L expression by CD8^+^ T cells confers protection against influenza infection by enhancing the early activation of CD8^+^ T cells.

### Expression of CD40L on CD8^+^ T Cells Enhances Their Secondary Expansion

**Figure 3:**
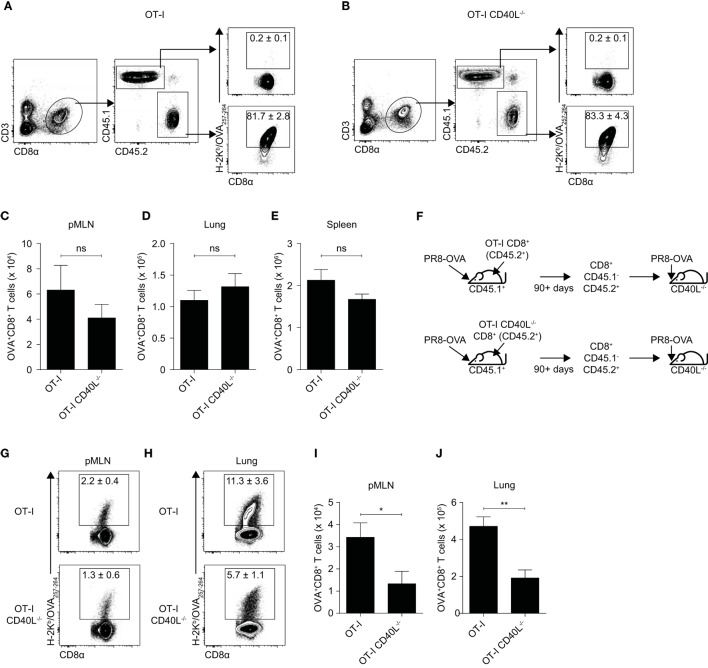
Expression of CD40L on CD8^+^ T cells enhances secondary expansion. **(A–E)** 10^5^ naïve OT-I or OT-I CD40L^−/−^CD8^+^ T cells (CD45.2^+^) were adoptively transferred into C57BL/6 (CD45.1^+^) recipients, and the hosts were infected with 50 PFU of PR8-OVA the following day. The **(A,B)** percentage and **(C–E)** number of donor (CD45.2^+^) OVA-specific CD8^+^ T cells in the **(C)** pMLN, **(D)** lung, and **(E)** spleen 90 days after infection were determined using flow cytometry. **(F–J)** CD45.1^−^CD45.2^+^CD8^+^ T cells were sorted from the spleens of the previously infected mice (90 days or more after infection), and 10^4^ sorted cells were adoptively transferred into naive CD40L^−/−^ mice. The mice were infected the following day with 50 PFU of PR8-OVA. **(G,H)** The percentage and **(I,J)** number of OVA-specific CD8^+^ T cells in the pMLN and lung 8 days after infection were measured by flow cytometry. Data are representative of two experiments and shown as mean ± SEM (*n* = 5 mice per group). Statistical analyses were performed using Student’s *t*-test (*p < 0.05, **p < 0.01). ns, not significant; pMLN, posterior mediastinal lymph node.

It has been proposed that CD8^+^ T cell-bound CD40L can also enhance their own secondary expansion during a *L. monocytogenes* infection (15) so we wanted to investigate whether this remains true during an infection with a virus such as influenza. Because the CD8^+^ T cell response in the prime-infect system we used was intended to mimic a secondary response, we hypothesized that CD40L is important for CD8^+^ T cells during a recall response during an influenza infection. We therefore infected C57BL/6 (CD45.1^+^) mice that received OT-I or OT-I CD40L^−/−^CD8^+^ T cells (CD45.2^+^) with PR8-OVA and rested them for at least 90 days. At this time point, most of the antigen-specific CD8^+^ T cells were derived from the transferred OT-I CD8^+^ T cells (Figures 3A,B) and there appeared to be no difference in the total number of memory CD8^+^ T cells between mice that received OT-I CD8^+^ T cells and those that received OT-I CD40L^−/−^CD8^+^ T cells (Figures 3C–E). To investigate the role of CD40L expression on the secondary expansion of CD8^+^ T cells, we sorted the transgenic CD8^+^ T cells based on CD45.2 expression and adoptively transferred them into CD40L^−/−^ hosts before infecting the hosts with PR8-OVA (Figure 3F). In this system, only the transferred CD8^+^ T cells were capable of CD40L expression. On day 7 after infection, the number of OVA-specific CD8^+^ T cells was determined in both the mediastinal lymph node (Figures 3G,I) and in the lung (Figures 3H,J). There was a greater than 50% reduction of expanded OVA-specific cells in mice that received memory OT-I CD40L^−/−^CD8^+^ T cells compared with those that received OT-I CD8^+^ T cells, suggesting a role for CD40L in the secondary expansion of CD8^+^ T cells.

**Figure 4:**
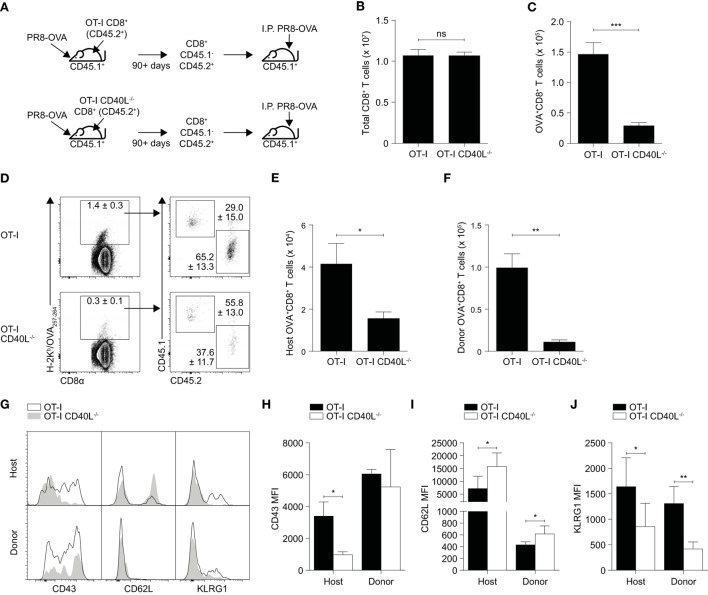
Bystander enhancement of CD8^+^ T cell expansion by expression of CD40L. **(A–J)** Memory CD45.1^−^CD45.2^+^CD8^+^ T cells were sorted from C57BL/6 (CD45.1^+^) mice that previously received OT-I or OT-I CD40L^−/−^CD8^+^ T cells (CD45.2^+^) and were infected with PR8-OVA (as described in Figure 3). 10^4^ sorted cells were adoptively transferred into naïve C57BL/6 (CD45.1^+^) mice, and the mice were infected intraperitoneally with 10^5^ PFU of PR8-OVA. The total number of **(B)** CD8^+^ T cells and **(C)** OVA-specific CD8^+^ T cells was determined by flow cytometry 8 days after infection. The OVA-specific CD8^+^ T cell response from the host (CD45.1^+^) and donor (CD45.2^+^) was also analyzed separately and shown as **(D)** percentages of the total OVA-specific CD8^+^ T cells and **(E,F)** absolute cell numbers. **(G–J)** The surface expression of CD43, CD62L, and KLRG1 on the host and donor OVA-specific CD8^+^ T cells was measured using flow cytometry and shown as **(G)** representative histograms and summarized in **(H–J)** their MFI. Data are representative of two experiments and shown as mean ± SEM (*n* = 4–5 mice per group). Statistical analyses performed using Student’s *t*-test (*p < 0.05, **p < 0.01, ***p < 0.001). MFI, mean fluorescence intensity.

Since antigen availability has been reported to affect the expansion and differentiation of CD8^+^ T cells (26, 27), variations in the rate of viral clearance may have contributed to the differences observed earlier. To rule that out, we repeated the experiment using an intraperitoneal route of infection which prohibits viral replication. As before, OT-I or OT-I CD40L^−/−^ resting OVA-specific memory CD8^+^ T cells were sorted based on CD45.2 expression, adoptively transferred into wild-type C57BL/6 mice (CD45.1^+^), and the mice were then infected intraperitoneally with PR8-OVA (Figure 4A). On day 7 after infection, although there was no difference in the total number of CD8^+^ T cells in the spleen of mice that received either OT-I or OT-I CD40L^−/−^CD8^+^ T cells (Figure 4B), only about 15% of the number of OVA-specific CD8^+^ T cells were found in mice that received OT-I CD40L^−/−^CD8^+^ T cells (Figure 4C), consistent with data from the previous experiment. Interestingly, we also found a decrease in the magnitude of the endogenous OVA-specific CD8^+^ T cell response in mice that received OT-I CD40L^−/−^ cells (Figures 4D–F), suggesting a cell-extrinsic effect of CD40L expression on the transferred memory CD8^+^ T cells. Differences in the expression of CD43, CD62L, and KLRG1 on both the endogenous and transferred OVA-specific CD8^+^ T cells were also observed between the two groups of mice (Figure 4G), with a greater proportion of cells from mice that received CD40L-competent cells being polarized toward the effector phenotype based on lower CD62L and higher KLRG1 expression, even in the endogenous OVA-specific cells (Figure 4H–J). Collectively, these data show that CD40L, when expressed on CD8^+^ T cells, promotes their own secondary expansion, and this effect is possibly cell extrinsic as bystander effects were observed.

### Expression of CD40L on CD8^+^ T Cells Promotes the Differentiation and Expansion of Effector CD8^+^ T Cells during *L. monocytogenes* Infection

To further investigate the mechanisms behind the function of CD8^+^ T cell-expressed CD40L, we decided to use a *L. monocytogenes* infection model for several reasons. First, it is possible that CD40L signaling may play a bigger role in infections by intracellular bacteria such as *L. monocytogenes* than in infections by viruses. CD40L signaling has been found to be a key process in regulating the secretion of IL-12 by DCs (13, 28), a pro-inflammatory cytokine involved in the induction of Th1 responses which is important in immune responses against intracellular pathogens. Since CD8^+^ T cell-expressed CD40L has the ability to induce the production of IL-12 p70 in DCs *in vitro* (13, 17), the expression of CD40L on CD8^+^ T cells may serve to enhance IL-12 production and Th1 induction. Second, the proportion of CD8^+^ T cells that express CD40L appears to be higher in the secondary lymphoid organs such as the spleen (Figure 1E), which is one of the primary sites of infection in the *L. monocytogenes* mouse model (29). Taken together, the *L. monocytogenes* model provides us with a system where the expression of CD40L on CD8^+^ T cells may be important.

**Figure 5:**
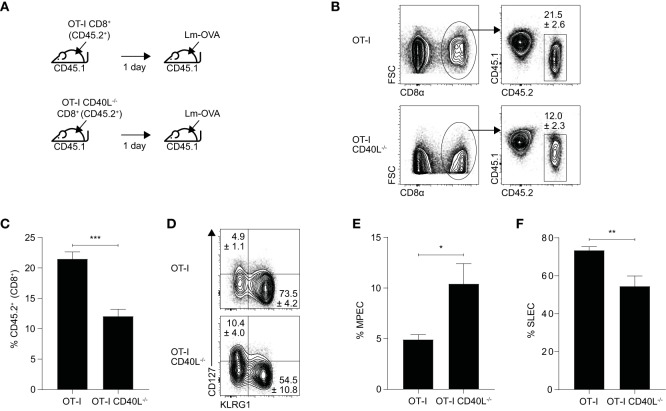
CD40L expression promotes the effector differentiation of CD8^+^ T cells during *Listeria* infection. **(A–F)** C57BL/6 (CD45.1^+^) mice received 10^3^ naïve OT-I or OT-I CD40L^−/−^CD8^+^ T cells (CD45.2^+^) and were infected with 10^4^ CFU of Lm-OVA the following day. **(B,C)** The number of donor (CD45.2^+^) CD8^+^ T cells was measured 7 days after infection, and **(D–F)** the proportion of MPECs and SLECs within the CD45.2^+^ donor population was determined by CD127 and KLRG1 staining. Data shown as mean ± SEM (*n* = 4–5 mice per group). Statistical analyses performed using Student’s *t*-test (*p < 0.05, **p < 0.01, ***p < 0.001). MPEC, memory precursor effector cell; SLEC, short-lived effector cell.

In this model (Figure 5A), naïve OT-I or OT-I CD40L^−/−^CD8^+^ T cells (CD45.2^+^) were sorted and adoptively transferred into wild-type C57BL/6 (CD45.1^+^) mice. The mice were infected intravenously the following day with a *L. monocytogenes* strain that expresses the OVA_257–264_ peptide (Lm-OVA). On day 7 after infection, the resulting OVA-specific CD8^+^ T cell response generated from the transferred cells was evaluated and as in the influenza model, the magnitude of the OVA-specific response was found to be lower in mice that received CD40L-deficient CD8^+^ T cells (Figures 5B,C). We also measured the expression levels of CD127 and KLRG1 and used them to determine the proportion of CD127^hi^KLRG1^lo^ memory precursor effector cells (MPECs) and CD127^lo^KLRG1^hi^ short-lived effector cells (SLECs) within the OVA-specific CD8^+^ T cell population (Figure 5D). We found that mice that received the CD40L-deficient cells had an increased proportion of MPECs and decreased proportion of SLECs compared with mice that received CD40L-competent cells (Figures 5E,F), consistent with previous results that showed an impairment in effector differentiation when the CD8^+^ T cells were not able to express CD40L.

### CD40L on CD8^+^ T Cells Enhances Their Own Proliferation in a Cell-Extrinsic Manner *via* Interactions with APCs

**Figure 6:**
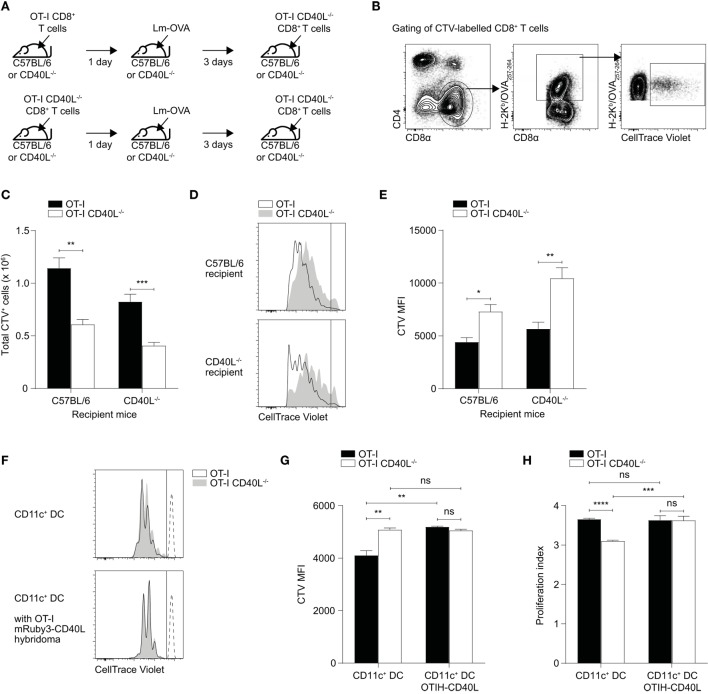
Cell-extrinsic enhancement of cell proliferation by CD40L-expressing CD8^+^ T cells. **(A–E)** 10^5^ naïve OT-I or OT-I CD40L^−/−^CD8^+^ T cells were adoptively transferred into C57BL/6 or CD40L^−/−^ recipient mice. The mice were rested for a day and then infected with 10^4^ CFU of Lm-OVA. After 3 days, 7.5 × 10^5^ CTV-labeled naïve OT-I CD40L^−/−^CD8^+^ T cells were adoptively transferred into the infected mice, and their spleens were harvested for analysis 3 days later (6 days after infection). For analysis, the CTV^+^ population was **(B)** gated from total splenocytes and **(C)** enumerated. **(D,E)** The magnitude of cell proliferation was determined by comparing the CTV MFI of CTV^+^ populations. **(F–H)** CTV-labeled naïve OT-I or OT-I CD40L^−/−^CD8^+^ T cells were cocultured with OVA_257–264_ peptide-pulsed CD11c^+^ dendritic cells (DCs) at a ratio of 3:1 (T cell:DC) in the absence or presence of OT-I T hybridoma cells that overexpress CD40L. The number of cell divisions as determined by CTV dilution was measured after 3 days of culture and shown as **(F)** representative histogram, **(G)** CTV MFI, and **(H)** proliferation index. Data shown as mean ± SEM [*n* = 4–5 mice per group for **(A–E)**; *n* = 3 replicates per group for **(F–H)**]. Statistical analyses performed using Student’s *t*-test (*p < 0.05, **p < 0.01, ***p < 0.001, ****p < 0.0001). ns, not significant; CTV, CellTrace Violet; MFI, mean fluorescence intensity.

Our observations thus far suggest that CD40L on CD8^+^ T cells may be acting in a cell-extrinsic manner and that the CD40L-deficient cells themselves are not inherently defective in proliferative potential or in effector capabilities. To test whether the presence of CD40L on CD8^+^ T cells can induce bystander effects on other CD8^+^ T cells, we adoptively transferred either OT-I or OT-I CD40L^−/−^ naïve CD8^+^ T cells into either C57BL/6 or CD40L^−/−^ mice and infected the mice the following day with Lm-OVA (Figure 6A). Three days after the infection, we labeled and transferred freshly isolated naïve OT-I CD40L^−/−^CD8^+^ T cells into the mice and waited another 3 days before recovering the spleens for analysis. In this system, the second batch of transferred CD8^+^ T cells was not able to express CD40L, and any source of CD40L signaling would be derived from either the host or the first batch of transferred cells. We enumerated the cells that were stained with CTV which represented cells from second transfer (Figures 6B,C) and found that there was a decrease in the number of these cells in mice that initially received CD40L-deficient cells. We then compared CTV dilution between the CTV^+^ populations and found that the cells that were recovered from mice that received CD40L-competent cells during the first transfer displayed a greater dilution in CTV compared with those recovered from mice that received CD40L-deficient cells (Figures 6D,E), consistent with the cell numbers observed. This suggests that the CD40L-expressing cells from the first transfer could create an environment that was more conducive for the second batch of cells to proliferate. The fact that this was observed in both the C57BL/6 and CD40L^−/−^ groups also provides evidence that this effect was due to the expression of CD40L on the transferred CD8^+^ T cells and not due to that of the endogenous cells.

As CD11c^+^ DCs are the primary APCs involved in the priming of CD8^+^ T cells in the spleen, we sought to test whether the interaction of CD40L-expressing CD8^+^ T cells with CD11c^+^ DCs could induce their activation and subsequently, improve the ability of the DCs to induce proliferation in CD8^+^ T cells. To this end, we sorted naïve OT-I and OT-I CD40L^−/−^CD8^+^ T cells, labeled them, and cocultured them *in vitro* with OVA_257–264_-pulsed CD11c^+^ DCs that were isolated from the spleen. In some samples, we added OT-I T hybridoma cells that were transduced with a lentiviral vector that induces the overexpression of mRuby3-tagged CD40L (Figure S3 in Supplementary Material) to serve as an exogenous source of CD8^+^ T cell-derived CD40L. Three days after the coculture, CTV dilution was measured using flow cytometry and we found that OT-I CD8^+^ T cells were able to proliferate more in comparison with OT-I CD40L^−/−^CD8^+^ T cells, with a substantial proportion of the CD40L-competent cells having undergone one more division than their CD40L-deficient counterparts (Figures 6F–H). This difference disappeared when CD40L-expressing OT-I T hybridoma cells were added to the culture, with the CD40L-deficient cells proliferating just as well as the CD40L-competent cells (Figures 6G,H), suggesting that the reduction in proliferative capacity exhibited by the OT-I CD40L^−/−^CD8^+^ T cells was not due to some inherent defect and that an exogenous source of CD40L could “rescue” them. Together with the data from the *in vivo* proliferation experiment, these observations suggest that CD40L-expressing CD8^+^ T cells were able to enhance—likely *via* APCs such as DCs—CD8^+^ T cell expansion in a cell-extrinsic manner.

## Discussion

Our results demonstrate that CD40L is upregulated substantially in a subset of CD8^+^ T cells and this allows CD8^+^ T cells to promote their own expansion and differentiation in a cell-extrinsic manner. Research dating from the 1990s has consistently shown that CD40–CD40L signaling is a key determinant of effective effector (1, 5, 7, 30) and memory (16, 31, 32) CD8^+^ T cell responses. The observation that CD8^+^ T cells are able to express CD40L themselves (33, 34) is thus intriguing. We found that the propensity of CD8^+^ T cells for CD40L expression depended on the localization of the CD8^+^ T cells as well as the environment in which they were primed, with the highest frequency of CD40L-expressing cells found in the secondary lymphoid organs. The localization of these cells in the secondary lymphoid organs where most of the T cell priming and differentiation take place suggest that CD8^+^ T cell-derived CD40L signals may be performing T cell “helper” functions such as DC activation. Some of the proposed functions of CD8^+^ T cell-expressed CD40L indeed include allowing CD8^+^ T cells to perform T-helper-like functions (14) and also to enhance their own secondary expansion (15). Here, we showed that CD8^+^ T cells that were primed in conditions where CD8^+^ T cell-derived CD40L signals were present were more strongly activated, able to proliferate better, and had improved cytotoxicity.

The idea that autologous signals allow CD8^+^ T cells to promote their own differentiation and expansion is not entirely new as it was reported that autocrine IL-2 is required for optimal secondary expansion of memory CD8^+^ T cells (20). Because the timely and efficient expansion and differentiation of either naïve or memory CD8^+^ T cells into cytotoxic effectors is often crucial in the greater context of cell-mediated immunity, the existence of these autologous and positive-feedback signaling pathways provides a mechanism by which to achieve such expansion. Indeed, CD8^+^ T cell-expressed CD40L itself is part of a positive-feedback loop involving APC-derived IL-12 (17, 34), where each promotes the expression and upregulation of the other.

Although reverse signaling through CD40L has been reported in the literature (35), our data show that CD8^+^ T cell-expressed CD40L is likely to act in a cell-extrinsic manner, as evidenced by our observation from the influenza model that adoptively transferred CD40L-expressing CD8^+^ T cells can promote the bystander activation and differentiation of endogenous CD8^+^ T cells. We also showed that CD40L-competent CD8^+^ T cells that were adoptively transferred into mice during a *L. monocytogenes* infection can enhance the proliferation of a separate group of CD40L-deficient CD8^+^ T cells that were transferred into the mice at a later time point. We propose that during the interaction of CD8^+^ T cells with DCs, CD40L allows the CD8^+^ T cells to activate CD40 on the DCs, inducing DC activation and “licensing.” This completes a positive-feedback loop where the outcome is enhanced CD8^+^ T cell activation, expansion, and differentiation. We have previously shown that CD40L on CD8^+^ T cells can induce the production of IL-12 in DCs (13), and others have reported that CD40L expression on CD8^+^ T cells can induce DC maturation and upregulation of costimulatory molecules such as CD80 and CD86 (36). CD40L signaling is also thought to be important in maintaining the cross-presentation capability of some DC subsets (31) and in the process of DC “reticulation” which significantly increases the cell surface area and spatial reach of DCs (37).

It remains possible that this proposed CD8^+^ T cell-DC positive-feedback loop also involves other molecules, as it was reported that IL-12 can prolong the division of activated CD8^+^ T cells by maintaining the expression of the high-affinity IL-2 receptor CD25 on the surface of CD8^+^ T cells (38). In this model, CD8^+^ T cells activate DCs *via* CD40L–CD40 interactions and induce the upregulation of IL-12 production, expression of costimulatory molecules such as CD80/86, and cross-presentation. The costimulatory molecules directly act on the CD8^+^ T cells to enhance differentiation and proliferation while IL-12 acts to upregulate CD25 and CD40L itself. This will complete the positive-feedback loop and simultaneously increase the sensitivity of the CD8^+^ T cells to autocrine IL-2 *via* CD25, the expression of which was often elevated in our experiments where the CD8^+^ T cells were CD40L competent.

Finally, our data do not completely rule out the relevance of T cell–T cell interactions where CD40–CD40L binding occurs directly between two CD8^+^ T cells and bypasses DC-bound CD40. However, this is unlikely to be the primary mechanism at play. While the expression of CD40 by CD8^+^ T cells has been shown to be relevant in T cell activation in some contexts (8, 19, 39), the transient nature of CD40L expression and its requirement for TCR engagement would mean that the direct activation of CD40 by CD40L expressed on another CD8^+^ T cells either requires the formation of a three-cell cluster of two CD8^+^ T cells and a DC or the direct recognition of a CD8^+^ T cell *via* TCR-cognate peptide MHC engagement by another CD8^+^ T cell. Whether the formation of three-cell clusters occurs at a frequency that makes it mechanistically relevant remains a matter of debate (40, 41) and the latter seems unlikely in our models where peptide-pulsed DCs or recombinant OVA-expressing bacteria and viruses whose antigens require processing and presentation by DCs were used. In addition, studies where infectious agents were used have shown that the expression of CD40 by CD8^+^ T cells was not critical for the development of functional CD8^+^ memory T cells (42, 43). Hence, although we do not dismiss the possibility of direct CD40–CD40L signaling between CD8^+^ T cells, we propose that CD8^+^ T cell-expressed CD40L acts primarily in a DC-dependent manner.

In summary, our data provide new insights into the role of CD40L when it is expressed on CD8^+^ T cells and suggest a mechanism where it allows CD8^+^ T cells to enhance their own proliferation and differentiation into effector cells.

## Ethics Statement

This study was carried out in accordance with the recommendations of institutional guidelines and was approved by the NUS Institutional Animal Care and Use Committee.

## Author Contributions

NT and DK conceived the study and designed the experiments. NT, DL, YC, and NP performed the experiments and analyses. NT interpreted the data and wrote the final manuscript, with critical input from DK and NG. DK and NG edited the manuscript and supervised the study. All the authors approved the final version.

## Conflict of Interest Statement

The authors declare that the research was conducted in the absence of any commercial or financial relationships that could be construed as a potential conflict of interest.
