# Supplementary material for: CD40L Expression Allows CD8+ T Cells to Promote Their Own Expansion and Differentiation through Dendritic Cells
Source: Front Immunol. 2017 Nov 6;8:1484. doi: 10.3389/fimmu.2017.01484 (PMC5672143; doi:10.3389/fimmu.2017.01484)
Supplement: Supplementary file 1 [file Image_1.PDF]

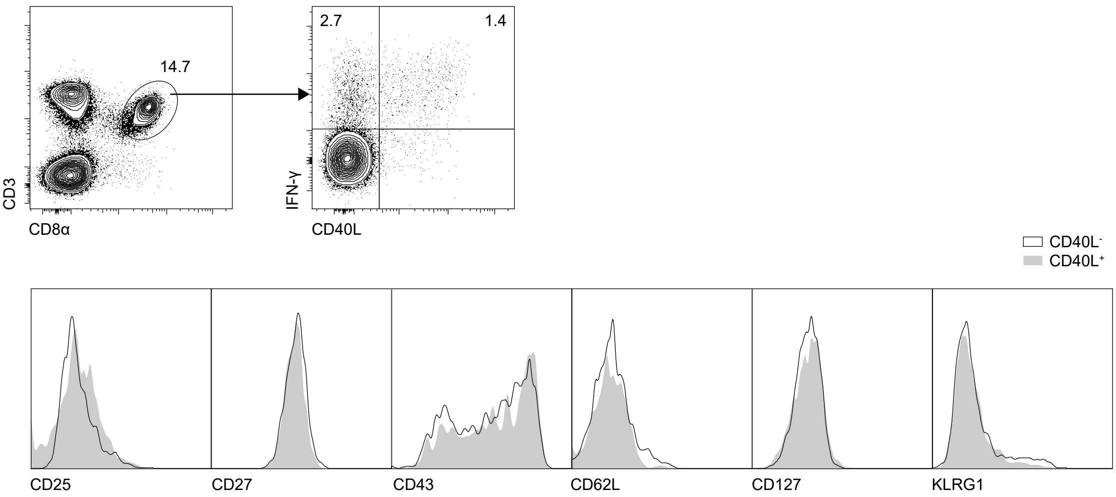

Supplementary figure 1. Phenotype of CD40L-expressing CD8$^+$ T cells. C57BL/6 mice were immunized with 5 × 10^5 DC-OVA. Splenocytes were harvested from the mice 6 days after immunization and restimulated *ex vivo* for 4 h with 1 µg/mL OVA$_{257-264}$. CD8$^+$ T cells were analyzed for IFN-γ and CD40L expression and the phenotypes of the IFN-γ$^+$CD40L$^-$ and IFN-γ$^+$CD40L$^+$ cells were compared on the basis of CD25, CD27, CD43, CD62L, CD127, and KLRG1 expression.

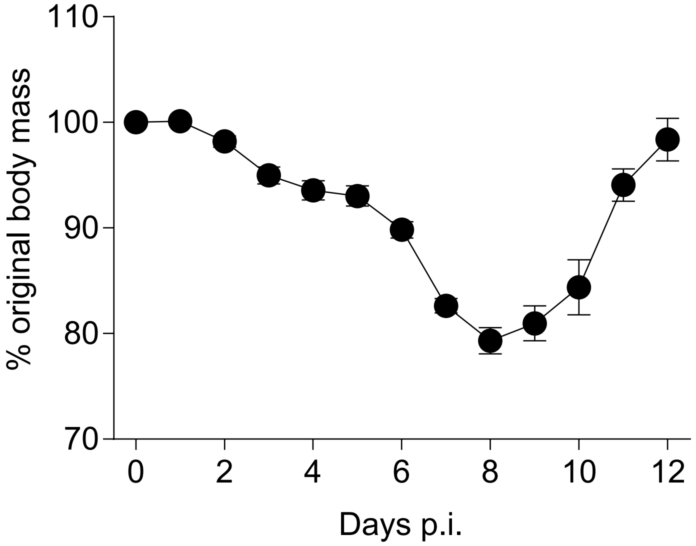

Supplementary figure 2. Infection of C57BL/6 mice with PR8-OVA. Weight change in C57BL/6 mice after infection with 50 PFU of PR8-OVA expressed as a percentage of original body mass.

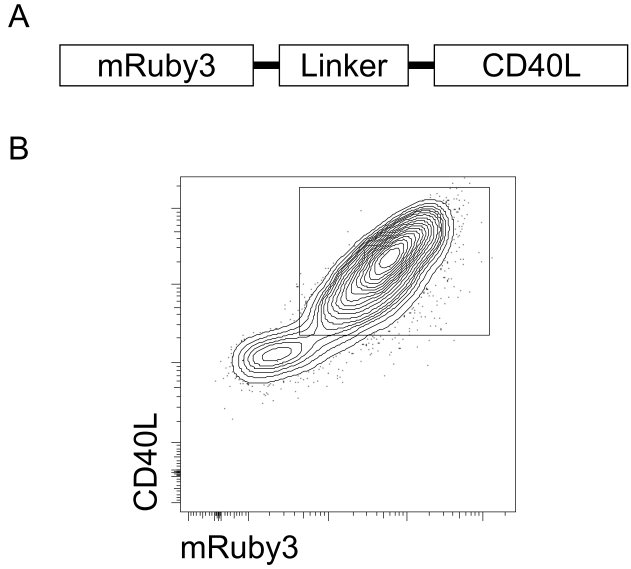

Supplementary figure 3. Overexpression of CD40L on OT-I T hybridoma by lentiviral transduction. (A) Murine CD40L was cloned using cDNA purified from activated OT-I CD8$^+$ T cells and fused to the C terminus of a red fluorescent protein (mRuby3) via a 15-aa flexible peptide linker (GGSGGGGSGGGGSGG). The mRuby3 in this construct localizes in the cytoplasmic region as CD40L is a type II transmembrane protein. (B) OT-I T hybridoma cells were transduced using a lentivirus packaged with the mRuby3-CD40L construct and were analyzed with flow cytometry for mRuby3 and surface CD40L expression.
